# Expression profiles of microRNAs from lactating and non-lactating bovine mammary glands and identification of miRNA related to lactation

**DOI:** 10.1186/1471-2164-13-731

**Published:** 2012-12-27

**Authors:** Zhen Li, Hongyun Liu, Xiaolu Jin, Lijan Lo, Jianxin Liu

**Affiliations:** 1 MOE Key Laboratory of Molecular Animal Nutrition, Hangzhou, 310058, PR China; 2 Laboratory of Functional Genomics, College of Animal Sciences, Zhejiang University, Hangzhou, 310058, P.R. China; 3 Institute of Dairy Science, College of Animal Sciences, Zhejiang University, Hangzhou, 310058, P.R. China

## Abstract

**Background:**

MicroRNAs (miRNAs) have been implicated in the regulation of milk protein synthesis and development of the mammary gland (MG). However, the specific functions of miRNAs in these regulations are not clear. Therefore, the elucidation of miRNA expression profiles in the MG is an important step towards understanding the mechanisms of lactogenesis.

**Results:**

Two miRNA libraries were constructed from MG tissues taken from a lactating and a non-lactating Holstein dairy cow, respectively, and the short RNA sequences (18–30 nt) in these libraries were sequenced by Solexa sequencing method. The libraries included 885 pre-miRNAs encoding for 921 miRNAs, of which 884 miRNAs were unique sequences and 544 (61.5%) were expressed in both periods. A custom-designed microarray assay was then performed to compare miRNA expression patterns in the MG of lactating and non-lactating dairy cows. A total of 56 miRNAs in the lactating MG showed significant differences in expression compared to non-lactating MG (*P*<0.05). Integrative miRNA target prediction and network analysis approaches were employed to construct an interaction network of lactation-related miRNAs and their putative targets. Using a cell-based model, six miRNAs (miR-125b, miR-141, miR-181a, miR-199b, miR-484 and miR-500) were studied to reveal their possible biological significance.

**Conclusion:**

Our study provides a broad view of the bovine MG miRNA expression profile characteristics. Eight hundred and eighty-four miRNAs were identified in bovine MG. Differences in types and expression levels of miRNAs were observed between lactating and non-lactating bovine MG. Systematic predictions aided in the identification of lactation-related miRNAs, providing insight into the types of miRNAs and their possible mechanisms in regulating lactation.

## Background

MicroRNAs (miRNAs) are small non-coding RNA molecules that are approximately 22 nucleotides (nt) in length, which negatively regulate specific target genes by mRNA degradation or translational repression. The role of miRNA was first reported in *Caenorhabditis elegans*, where aberrant expression of *lin-4* caused abnormal cell division and proliferation, affecting the timing of cell division and development in larvae [1]. Several developmental and physiological processes including stem cell differentiation, hematopoiesis, cardiac and skeletal muscle development, neurogenesis, insulin secretion, cholesterol metabolism and immune response have been shown to be regulated by miRNAs [2]. The expression of most miRNAs has a spatio-temporal pattern, suggesting that they play specific functions in a variety of processes. Recently, several studies have explored miRNAs as molecular biomarkers for use in identifying biological pathways, aiding cancer diagnoses, and identifying disease activity and treatment effects [3,4].

The bovine mammary gland (MG) is a complex organ that grows and develops after calf birth [5]. The complex initiation of MG lactation has been extensively studied over the years at the genetic, physiological and morphological levels because of its important functions [6]. It has been reported that many genes are expressed differently to maintain lactation [7-9]. However, only a few studies have assessed the potential implication of miRNAs in MG lactogenesis. Numerous miRNAs have been found to be involved in the regulation of milk protein expression and MG differentiation, and computational and experimental methods have been exploited to identify miRNAs in cattle [10]. However, studies on the regulation of miRNA expression profiles in bovine MG during lactation are still in their infancy. There are currently 730 bovine miRNAs deposited in miRbase 17.0 [11], with only a few found in the MG [12,13]. Therefore, identifying MG miRNA expression profiles is an important approach to explore the mechanism of lactation initiation and to identify biomarkers for lactogenesis.

To obtain miRNA expression profiles and to compare the difference in miRNA expression between periods of lactation and non-lactation, we used next-generation sequencing technology to sequence two miRNA libraries constructed from tissue samples taken during these two periods. Using computational prediction, potential targets for these miRNAs were identified, leading to the construction of an interaction network related to lactation. Our integrative analysis highlights the complexity of gene expression networks regulated by miRNAs in MG during lactation.

## Results

### Determination of bovine MG period

**Figure 1:**
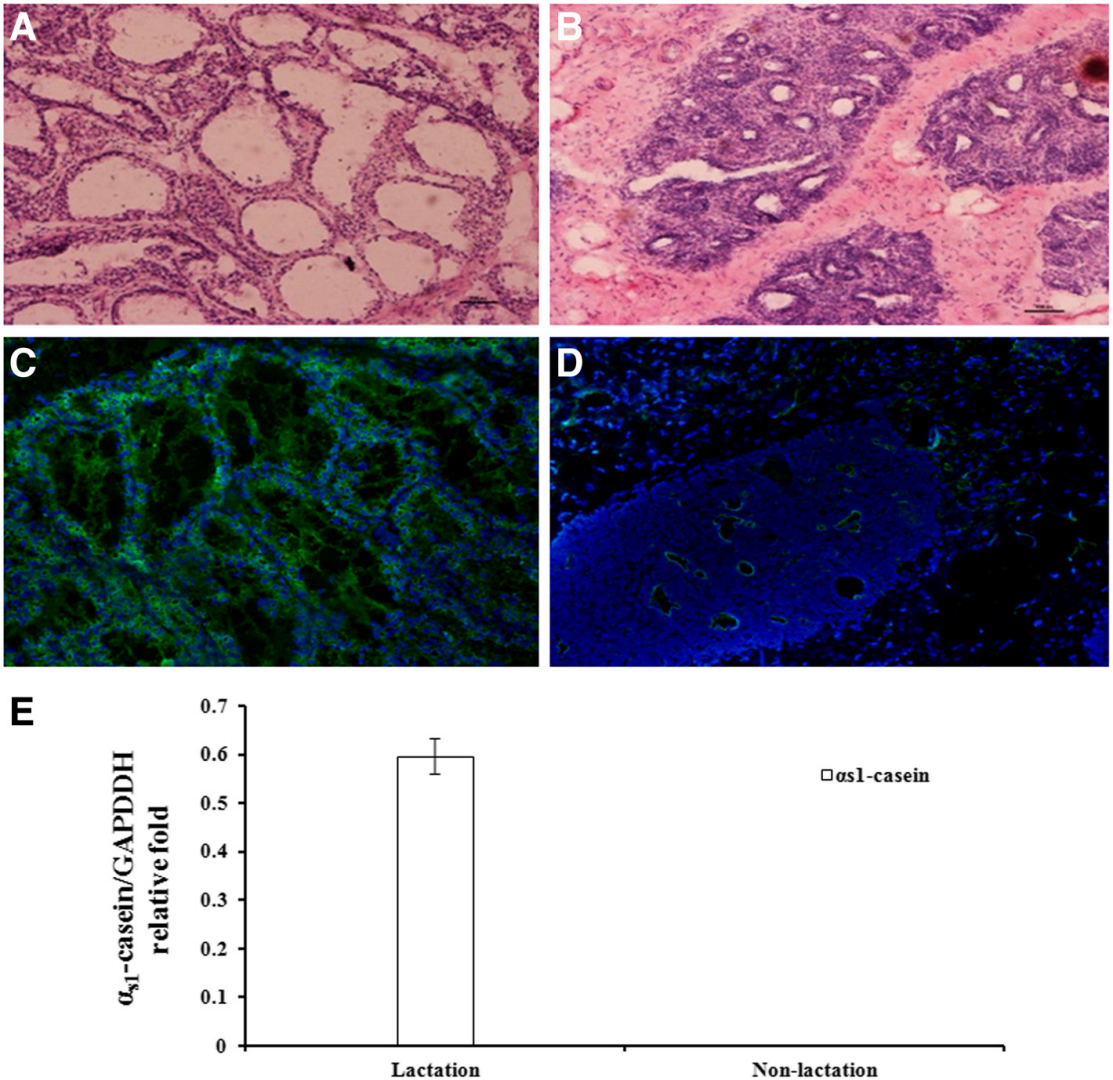
**The microstructure and gene expression of lactating and non-lactating mammary gland tissue in the dairy cow. (A)** Paraffin sections of bovine mammary gland in the lactation period (100×). **(B)** Paraffin sections of bovine mammary gland in the non-lactation period (100×). In **(A)** and **(B)**, the MG sections were stained with hematoxylin-eosin using standard procedures. Nuclei are dyed blue by hematoxylin, and the cytoplasm is stained pink by eosin. Hematoxylin-eosin stained sections were analyzed using light microscopy. **(C)** Expression of α-casein in lactating bovine mammary glands (100×). **(D)** Expression of α-casein in non-lactating bovine mammary glands (100×). In **(C)** and **(D)**, nuclei are in blue as marked by DAPI, and the α-casein signal is in green. Alpha-casein was detected using immunofluorescence using an anti-α-casein antibody followed by HRP-conjugated goat anti-rabbit IgG and analysis using fluorescence microscopy. **(E)** Expression of α_s1_-casein mRNA in lactating and non-lactating bovine mammary glands using real-time PCR.

Hematoxylin-eosin staining (HE) and immunofluorescence (IF) were employed to verify the microstructure differences of the lactating and non-lactating MG tissues used in constructing miRNA libraries. In the lactation MG (Figure 1A, 1C), many mature alveolar structures were packed with mammary lobules of a variety of shapes. Mature alveolar lumens were large in appearance and filled with secretions, with little connective tissue between alveoli. Additionally, large amounts of α-casein were found surrounding the nuclei and in the large alveolus. In contrast, an increase in stromal, connective and fatty tissue was observed in the non-lactating MG (Figure 1B, 1D). In addition, most of the ductal lumens were either comparatively smaller than in the lactating MG or collapsed, with a few residual ductal forming clusters of epithelial cells. Small amounts of α-casein were detected around the tissues.

Furthermore, mRNA expression of α_s1_-casein, a major milk protein, was measured using real-time PCR. As expected, α_s1_-casein mRNA was highly expressed during the lactation period and had barely detectable expression during the non-lactation period (Figure 1E).

### Analysis of sequencing data

Two miRNA libraries were constructed using small RNA isolated from bovine MG and sequenced using Genome Analyzer. A total of 15,089,573 and 18,079,366 reads were obtained from the lactation and non-lactation period libraries, respectively (Table 1). The ratio of lactation/non-lactation was 83.5%, indicating that the two libraries were well represented.

**Table 1:** Statistics based on the reads of the sequencing data

	**Average reads**	**Lactation period**	**Non-lactation period**
		**reads**	**reads**
total reads	16,584,470	15,089,573	18,079,366
raw reads	15,389,545	13,711,046	17,068,043
clean reads	13,966,513	11,964,909	15,968,116
known miRNA	12,387,809	10,231,155	14,544,463
conserved miRNA	83,064	76,098	90,030
novel miRNA	164,893	88,088	241,697

**Figure 2:**
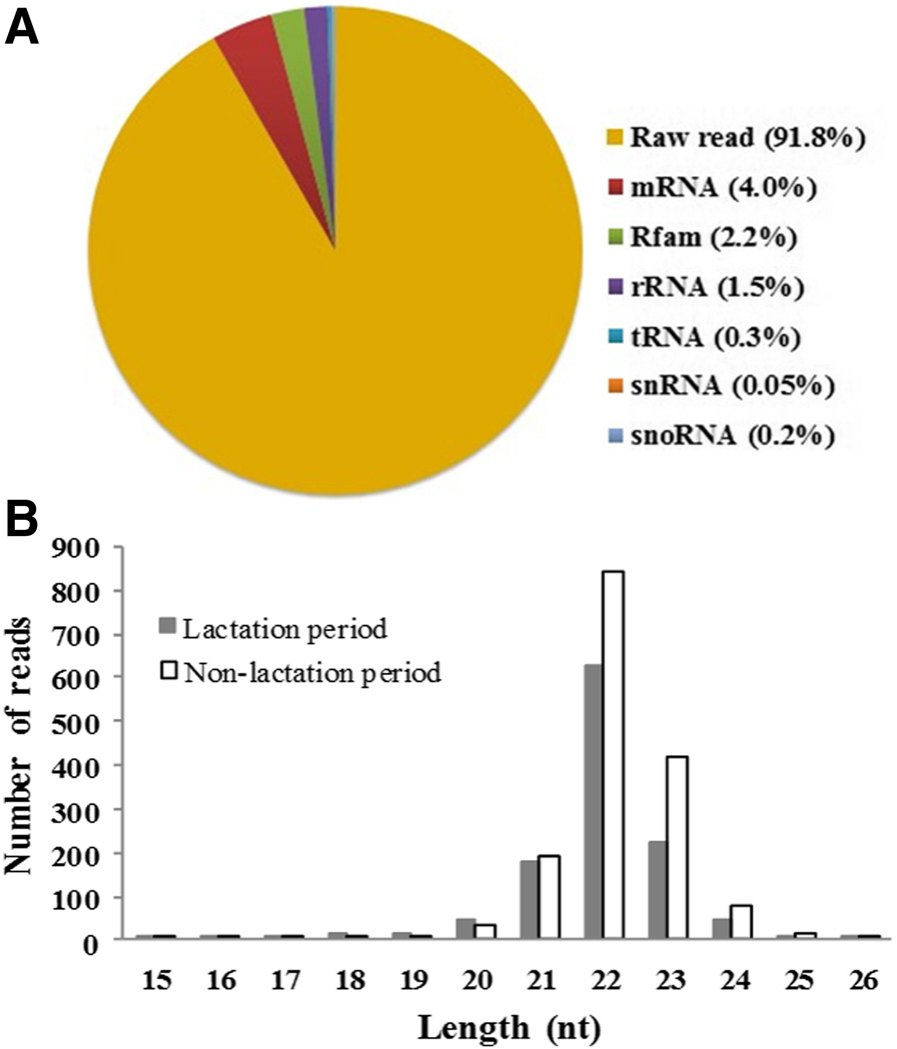
**The characteristics of sequencing reads. (A)** Count number distribution of the various RNA classes. **(B)** Length distribution of small RNAs of various lengths (15–26 nt).

After filtering low-quality reads and adaptor sequences, 13,711,046 raw reads were obtained from the lactation library and 17,068,043 from the non-lactation library (Table 1). Certain RNA reference sequences including mRNA, rRNA, tRNA, snRNA, snoRNA and Rfam were filtered from the raw reads. The detection of a low proportion of long RNAs, such as mRNA (4.2%), rRNA (1.6%), tRNA (0.3%) and snoRNA (0.2%) (Figure 2A), indicated that our samples were not contaminated by degraded RNA. Finally, 11,964,909 and 15,968,116 clean reads from lactation and non-lactation libraries, respectively, were used for subsequent analysis (Table 1). Length distribution analysis revealed that more than half of these clean reads (52.6%) were 22 nt in length (Figure 2B), consistent with the typical size range of small RNA generated by Dicer. This analysis indicated that the clean reads included a large proportion of miRNA sequences.

All clean reads were then aligned against the *bta* genome and mammalian miRbase using the ACGT-miR program. This process detected 885 pre-miRNAs encoding 921 miRNAs, of which 884 were unique sequences, while 544 (61.5%) were expressed in both periods. The unique miRNAs were categorized into three groups based on their hits: 283 miRNAs matched with known *bta* miRNAs registered in the miRbase database; 96 miRNAs were conserved among other mammals but have yet to be identified in bovines; and 505 miRNAs were mapped to the *bta* genome, with the extended genome sequences having the potential to form hairpins.

### Features of the three miRNA categories in bovine mammary glands

**Figure 3:**
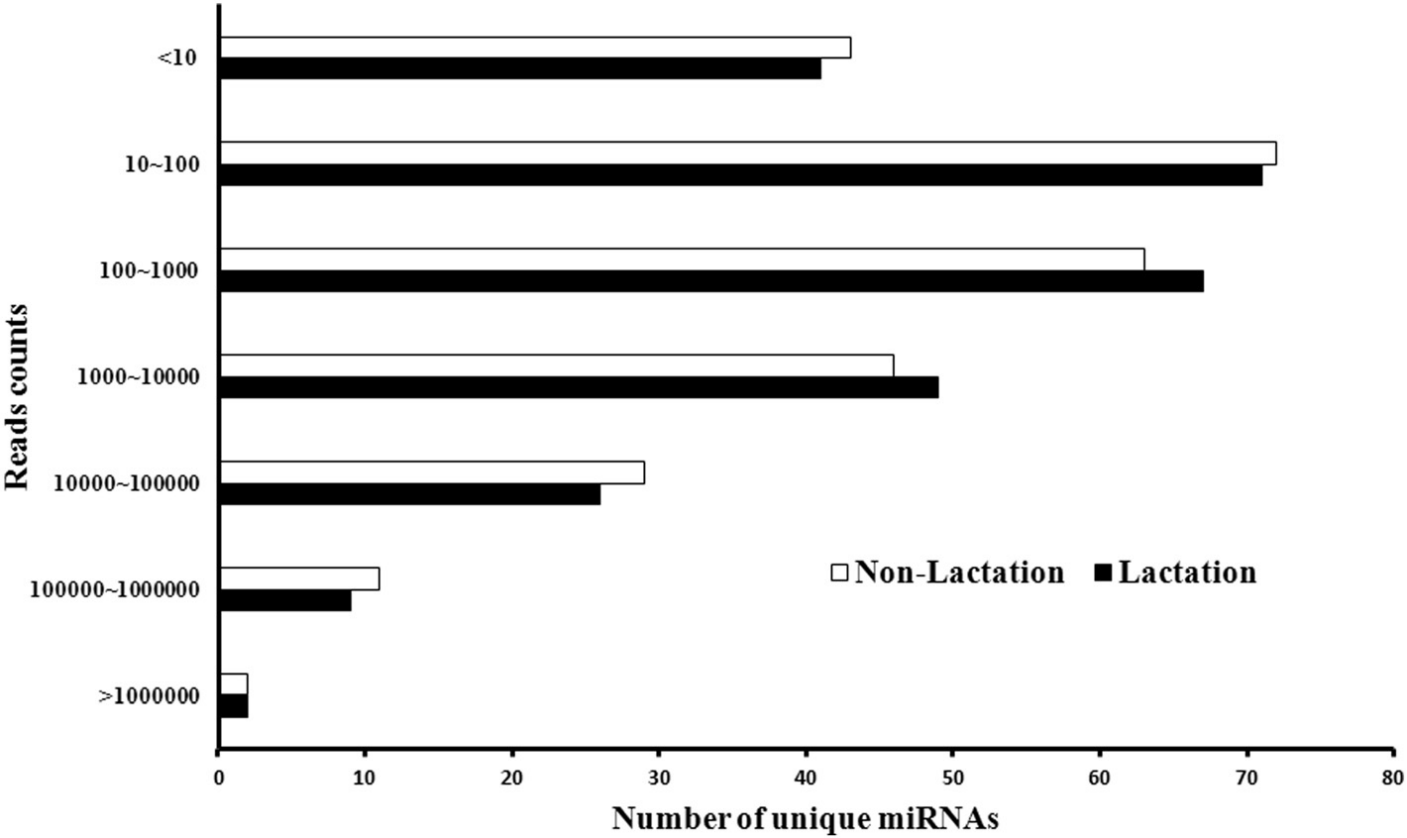
**The distribution of the sequencing reads of known bovine miRNAs.** The read counts of known bovine miRNAs in lactating and non-lactating mammary glands were classified. The known miRNAs had a broad range of expression levels in the MG, ranging from hundreds of thousands of sequence reads for the most abundant miRNAs to single reads for the least abundant.

The 283 unique sequences from 298 known miRNAs corresponding to 273 *bta* pre-miRNAs only accounted for 38.8% (283/730) of the sequences deposited in miRbase (version 17.0) (Additional file 1). Reads of these known miRNAs accounted for 88.7% (12,387,809/13,966,513) of the total clean reads (Table 1). These results indicated that while known, highly abundant *bta* miRNAs were easily detected, there are novel miRNAs in the MG that have yet to be isolated. The known miRNAs had a broad range of expression levels in the MG, ranging from hundreds of thousands of sequence reads for the most abundant miRNAs to single reads for the least abundant (Figure 3). The two libraries had 248 (87.6%) of the known miRNAs in common, indicating that most of the known miRNAs were shared during MG development.

**Figure 4:**
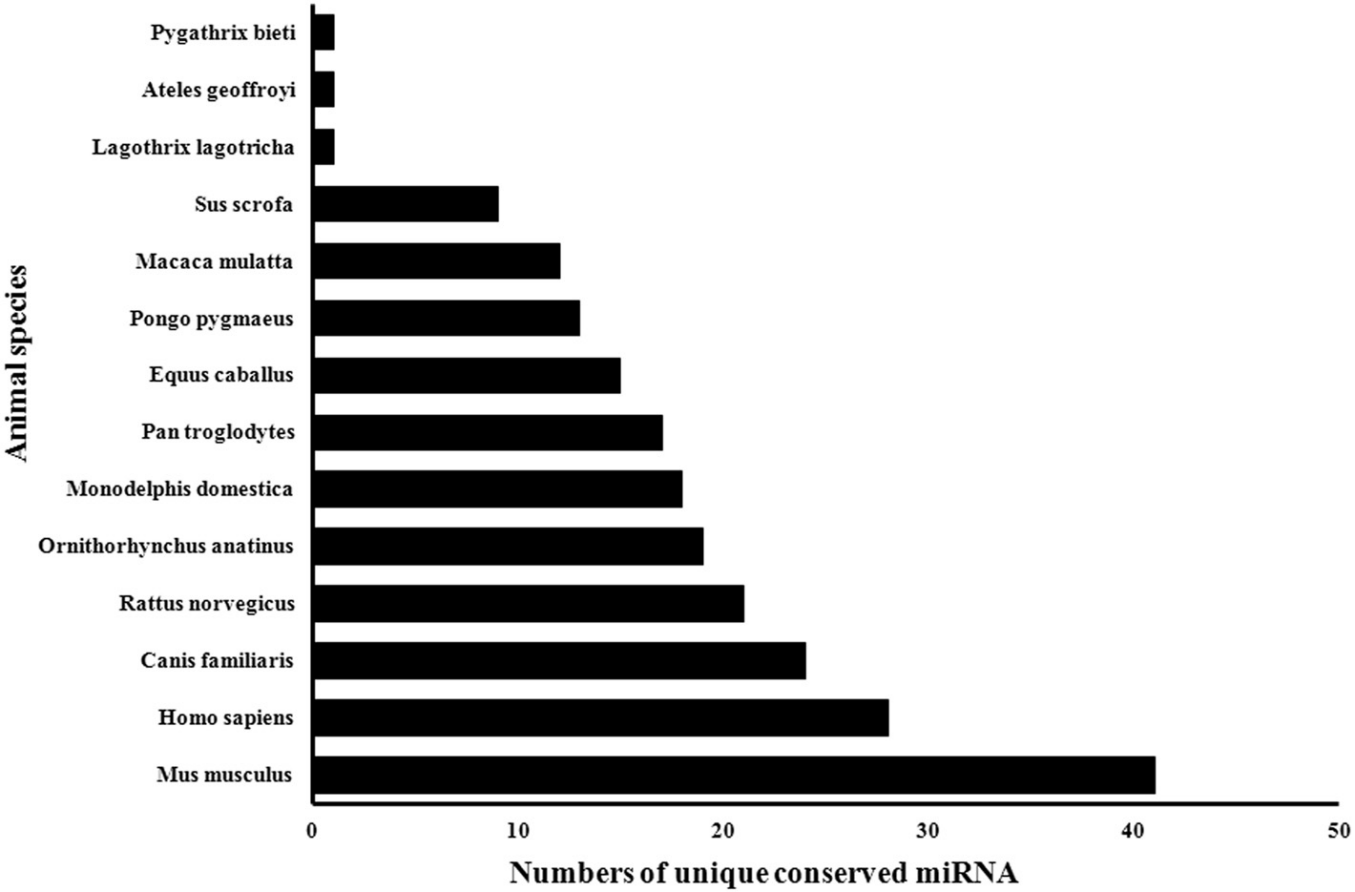
**The conservation profile of conserved bovine miRNAs.** These sequences were mapped to other mammalian genome assemblies and shared highly similar sequences. Values on the X axis indicate the number of unique conserved miRNAs between *bta* and the queried species.

The sequences were also mapped to other mammalian genomes. In this study, the 106 miRNAs prefixed with “PC” corresponding to 96 unique miRNA sequences were classified as conserved (Additional file 2). Our results showed that 106 genomic cognates were predicted to be homologous miRNAs, of which 41 were cognates in *Mus musculus*, 28 in *Homo sapiens*, 24 in *Canis familiaris* and 21 in *Rattus norvegicus* (Figure 4). In general, the bovine miRNA population is more conserved in *Mus musculus* than in *Homo sapiens*.

The 517 miRNAs representing 505 unique miRNA sequences comprising the third group were termed as novel bovine miRNAs and prefixed with “CN” because they mapped to the *bta* genome without homology to any known mammalian miRNAs, and the corresponding extended genome sequences are capable of forming hairpins (Additional file 3). This miRNAs in this group accounted for a large proportion of the total expressed unique miRNAs (more than 50%, 505/884) and were novel miRNAs in cattle. In this unique “CN” series of miRNAs, 239 (239 out of 505, 43.9%) novel bovine miRNAs were detected in both libraries, while the remaining miRNAs were expressed in a lactation period-specific manner. Unsurprisingly, the reads of novel bovine miRNAs were less abundant than those of known miRNAs. Only 6 and 7 novel bovine miRNAs produced more than 1,000 reads in the lactation and non-lactation libraries, respectively.

A custom-designed microarray assay was performed to validate these miRNA expressions in the MG of lactation and non-lactation dairy cows. After removing miRNAs that had less than 10 reads (as a cutoff value) from the original 884 unique miRNAs, the remaining 523 miRNAs (283 known, 51 conserved and 189 novel) were fabricated on the microarray. Only 304 of the 523 miRNAs were identified by the microarray, including 187 of the known miRNAs, 43 of the conserved miRNAs and 74 of the novel miRNAs (Additional file 4).

### Identification of differential expression patterns of miRNA in bovine MG

**Figure 5:**
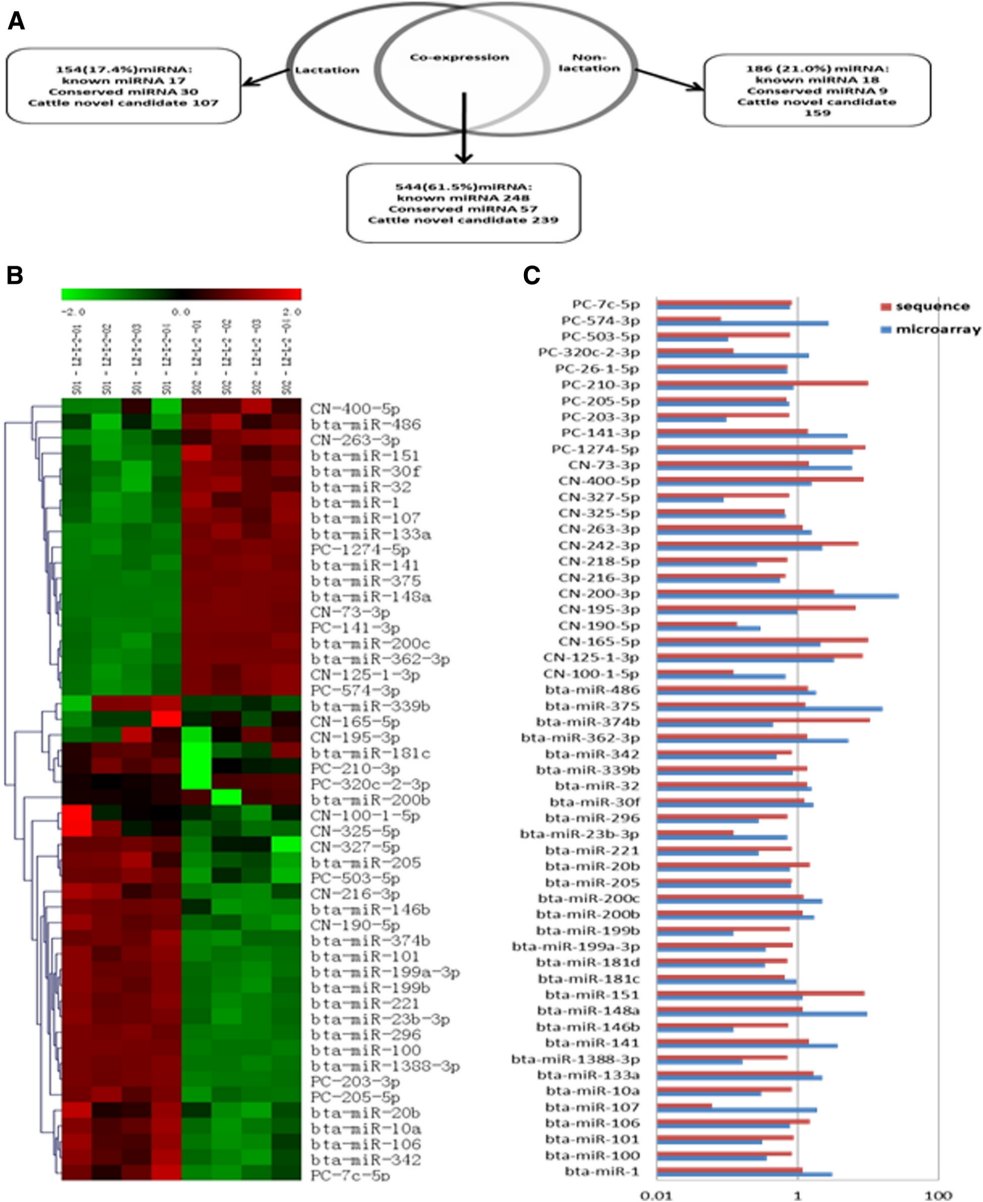
**Differential miRNA expression in the mammary gland. (A)** The Venn diagram displays the distribution of 884 unique miRNAs found in lactation and non-lactation periods. **(B)** The heat map was constructed based on the mean expression levels of the microarray. **(C)** Comparison between the microarray (blue bar) and sequencing (red bar) data. The bar graph shows the ratio of lactation to non-lactation (R value), where R values of higher or lower than 1 in both methods indicate that the miRNA has the same expression trend in the two different assay systems. R values where R_(microarray)_>1 and R_(sequencing)_<1 indicate that miRNA changes are differently represented by the two methods.

The main aim of this study was to identify miRNAs that may play regulatory roles in bovine MG by comparing their expression patterns in lactation and non-lactation periods. We examined the co-expression of unique miRNAs in the two libraries representing these two periods (Figure 5A). Analysis of the sequencing results demonstrated that more than 60% of reads overlapped between lactation and non-lactation periods, in which 248 miRNAs were known, 57 miRNAs were conserved and 239 were novel. The IDEG package was then used to identify differentially expressed miRNAs on the basis of potentially significant changes in expression. The expressions of 56 miRNAs were significantly different between lactation and non-lactation periods (*P*<0.05, Additional file 5). Among these, 41 unique miRNAs were expressed in both periods, while 9 miRNAs (mir-151-3p, mir-374b-5p, PC-1274-5p, PC-210-3p, CN-125-1-3p, CN-165-5p, CN-195-3p, CN-242-3p and CN-400-5p) were only highly expressed in the lactation period and 6 miRNAs (mir-107-3p, mir-23b-3p, PC-320c-2-3p, PC-574-3p, CN-100-1-5p and CN-190-5p) were only highly expressed in the non-lactation period. Microarrays were performed to confirm these 56 differentially expressed miRNAs. The expression patterns of 48 of these miRNAs (48/56, 85.7%) were consistent with the deep sequencing data (Figure 5B, 4C). This finding indicates that deep sequencing is slightly more sensitive and reliable for the identification of differentially expressed miRNAs than microarray analysis.

### Characteristics of chromosomal locations of pre-miRNAs in bovine mammary glands

The chromosomal positions (*bta* genome assembly) of all 885 pre-miRNAs detected in this experiment, including novel miRNAs, were searched by BLAST. It was determined that 800 of the pre-miRNAs (90.4%) matched with the *bta* genome and that 26 mature miRNAs of pre-miRNA hairpins were located at more than two genomic loci on different chromosomes (Additional file 6).

**Figure 6:**
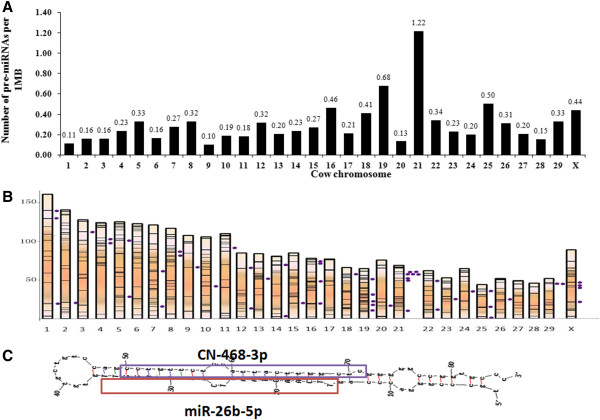
**Features of chromosomal locations of pre-miRNA in bovine MG. (A)** Pre-miRNA densities on bovine chromosomes, calculated by the number of pre-miRNAs per Mb of DNA. **(B)** The locations of bovine pre-miRNAs on each chromosome. Dots represent 55 clusters; black lines represent pre-miRNA locations. **(C)** The pre-miRNA structure. The mir-26b-5p mature sequence is boxed in red, while the CN-468-3p sequence is boxed in purple.

The genomic density distribution of bovine pre-miRNAs, i.e., the number of pre-miRNAs per Mbp of individual chromosome, was analyzed (Figure 6A). Densities were calculated by dividing the number of pre-miRNAs by the number of nucleotides on the individual chromosome. The shortest chromosome (25 with 44 Mbp) and the longest chromosome (1 with 161 Mbp) encode 22 and 18 pre-miRNAs, respectively, corresponding to 0.50 and 0.11 pre-miRNAs per 1 Mbp genomic sequence. Chromosome 21 encodes 84 pre-miRNAs, the most pre-miRNAs of all the chromosomes, corresponding to 1.22 pre-miRNAs per 1 Mbp genomic sequence. The coefficients of variation (CV) are 72% in this study and 73.6% as reported in miRbase [14], indicating a consistent genomic density distribution (r=0.953, *p*<0.01).

In gene studies, genes are clustered to identify co-expressed genes from the same primary transcript or to identify gene clusters that share similar functions. We followed the criteria proposed by miRbase and defined 10 Kbp as the maximum inter-distance for two pre-miRNAs to be considered as clustered. There were 230 pre-miRNAs grouped into 55 clusters, accounting for only 28.75% (230 out of 800) of the total pre-miRNAs (Figure 6B).

We predicted all of our mature miRNAs of pre-miRNA hairpins using UNAfold software and found that 104 pairs of known miRNAs and novel candidates share the same pre-miRNA structure and that their pre-miRNAs’ chromosomal locations were identical (Additional file 7). The two mature sequences were located in different arms of one pre-miRNA, including 39 known miRNAs located at the 3’ end and 65 known miRNAs at the 5’ end. We also found that reads of sequences were heavily biased towards the arm containing known miRNAs. For example, mir-26b-5p and PC-468-3p share the same hairpin structure and are located on different sides of chromosome 2 (genomic location of pre-miRNA: 110812687–110812771) (Figure 6C); the reads of each sequence for mir-26b-5p and PC-468-3p were 108,006 and 7 in the lactation period and 118,498 and 8 in the non-lactation period, respectively. It is possible that these lower-read novel candidates may be new miRNAs revealed by the highly sensitive deep sequencing approach.

### Prediction of miRNA targets and lactation networks in bovine MG

**Figure 7:**
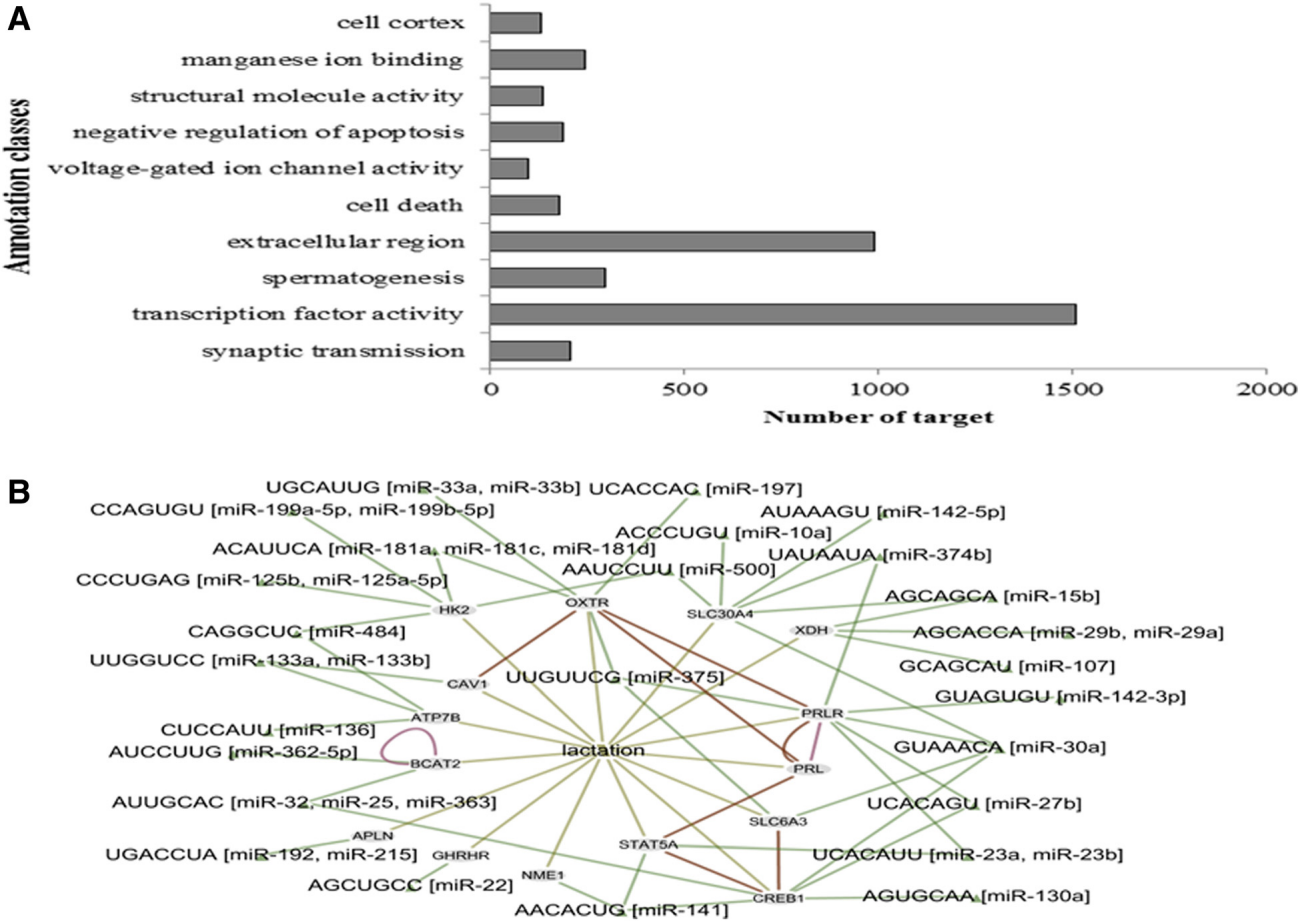
**Prediction of miRNA targets and lactation network. (A)** Gene ontology annotation analysis of miRNA targets. **(B)** The constructed lactation network of miRNAs. In the network, miRNAs are represented by triangles, and target genes are represented by grey nodes. Green lines represent miRNA-target interactions, brown lines denote gene-gene pathway interactions, and pink lines represent the NHGRI GWA catalog.

The 283 known bovine miRNAs detected were grouped into families with unique seed sequences (2–8 nt) to identify putative targets. More than 10,000 annotated mRNA transcripts were selected as potential targets, equivalent to approximately 35 targets per miRNA. All targets were then processed by Gene Ontology annotation analysis. It was found that these targets have a wide range of diverse functions, with over half involved in transcriptional activity (Figure 7A).

Based on the results of target prediction, miRNAs and target gene interactions were integrated using EGAN to construct possible regulatory networks to investigate the relationship between miRNAs and lactation (Figure 7B). We chose lactation as the Gene Ontology central process to show all Entrez gene neighbors and then imported 283 known miRNAs to find possible relationships. The resulting network includes 37 miRNAs detected by this study and 15 expressed target genes relating to lactation. For example, miR-138, reported to have a role in lactation, has five predicted targets related to lactation, including *SLC35b2*, *CABP4*, *GPR*, *MAPKAP* and *PRLR*. Of these predicted targets, miR-138 is known to inhibit PRLR protein translation by regulating *STAT5* and *MAPK*, thereby suppressing the proliferation and viability of mouse mammary epithelial cells [15]. In this study, miR-138 was expressed more highly during lactation than during non-lactation, as detected by deep sequencing. Prolactin, which acts through its receptor, is a key factor in lactogenesis and lactation maintenance [16]. *PRLR* causes ductal outgrowth and side branching when grafted into *PRLR*^−/−^ epithelium [17].

### Regulation of lactation genes by miRNAs

**Figure 8:**
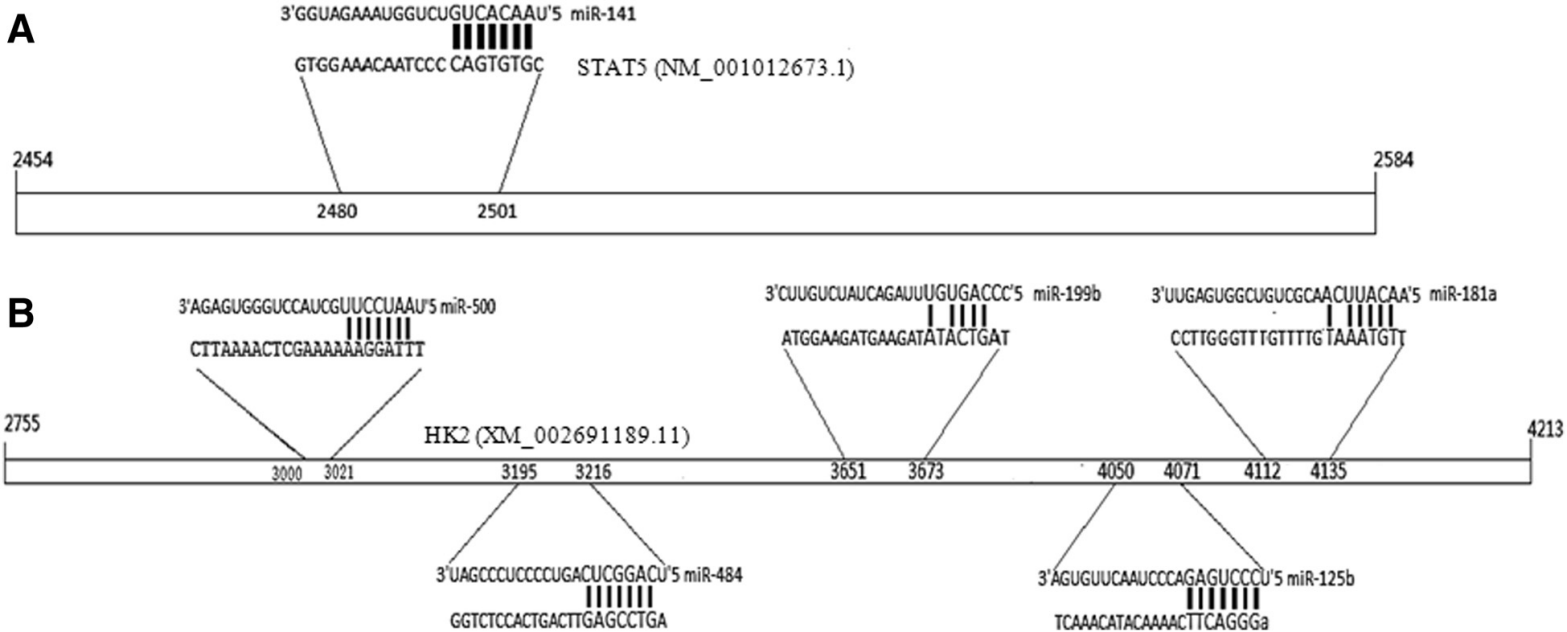
**Putative miRNAs targeting gene 3’UTRs. (A)** Pairing of bta-miR-141 with the bovine *STAT5* 3’UTR sequence. **(B)** A target seed region of the miRNA binding site was found within the bovine *HK2* 3’UTR sequence.

Two genes and six miRNAs in the network were selected to validate its reliability. Our data collection and analysis revealed that the 3’UTR of signal transducers and activators of transcription (*STAT5*, NM_001012673.1) mRNA contains a complementary site for the seed region of bta-miR-141, while the 3’UTR of Hexokinases (*HK2*, XM_002691189.1) was paired with five miRNAs: bta-miR-500, bta-miR-199a, bta-miR-125b, bta-miR-181a and bta-miR-484 (Figure 8). These findings highlight the fact that our network is able to propose possible interactions between miRNAs and genes that have yet to be determined experimentally.

**Figure 9:**
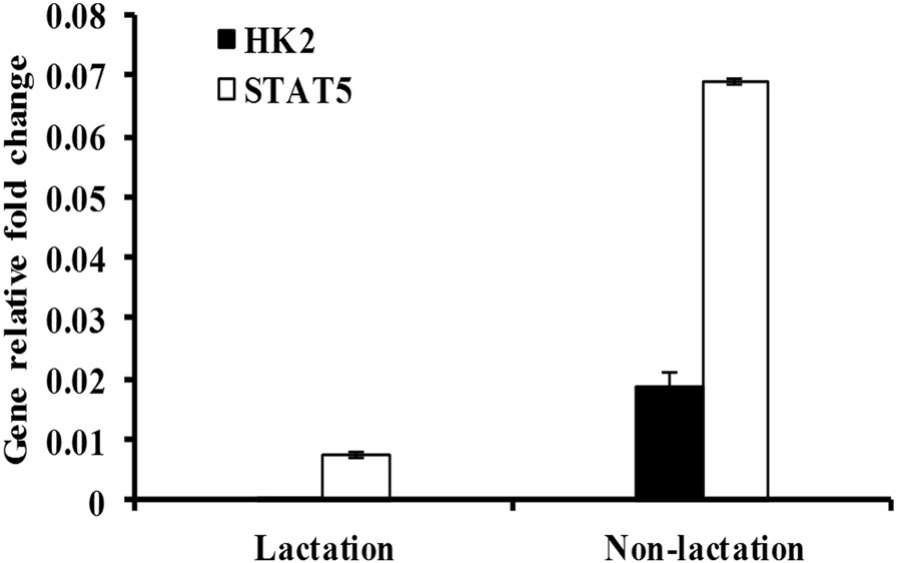
**Expression of *HK2 *and *STAT5 *mRNA in the bovine mammary gland.** Expression levels of *HK2* and *STAT5* in lactating and non-lactating bovine mammary glands were determined using real-time PCR. Gene fold changes were calculated using the 2^−ΔCt^ method, where ΔCt = (Ct _HK2/STAT5_ − Ct _GAPDH_). n=3.

Real-time PCR analysis was performed to analyze the expression levels of miR-141, miR-125b, miR-181a, miR-199b, miR-484 and miR-500 in MG tissues. MiR-141, miR-484 and miR-500 were readily detectable in both periods but were expressed at obviously lower levels in the lactation period than in the non-lactation period. In marked contrast, significant reductions in the expression of miR-125b, miR-181a and miR-199b were observed in the non-lactation period (Table 2). Expression of *HK2* and *STAT5* mRNA was found to be higher in the non-lactation period than in the lactation period (Figure 9).

**Table 2:** MiRNA mammary gland expression

**Assay Name**	**Fold Change (L/NL)**		
	**compared with non-lactation**		
	**sequence**	**microarray**	**real-time PCR**
miR-125b	0.26	0.53	0.61
miR-141	12.82	19.09	37.25
miR-181a	0.48	0.85	0.08
miR-199b	0.20	0.12	0.38
miR-484	3.41	1.97	295.83
miR-500	3.97	2.98	334.56

The significant differences in the expressions of miRNAs and their targets between the two periods prompted us to explore the possible biological significance. We investigated the possible roles of these differentially expressed miRNAs in the regulation of gene expression using a cell-based model. MiR-141 mimic, miR-141 inhibitor, miR-181 mimic, miR-199a mimic, miR-484 mimic and miR-500 mimic were individually transfected into Mac-T cells.

**Figure 10:**
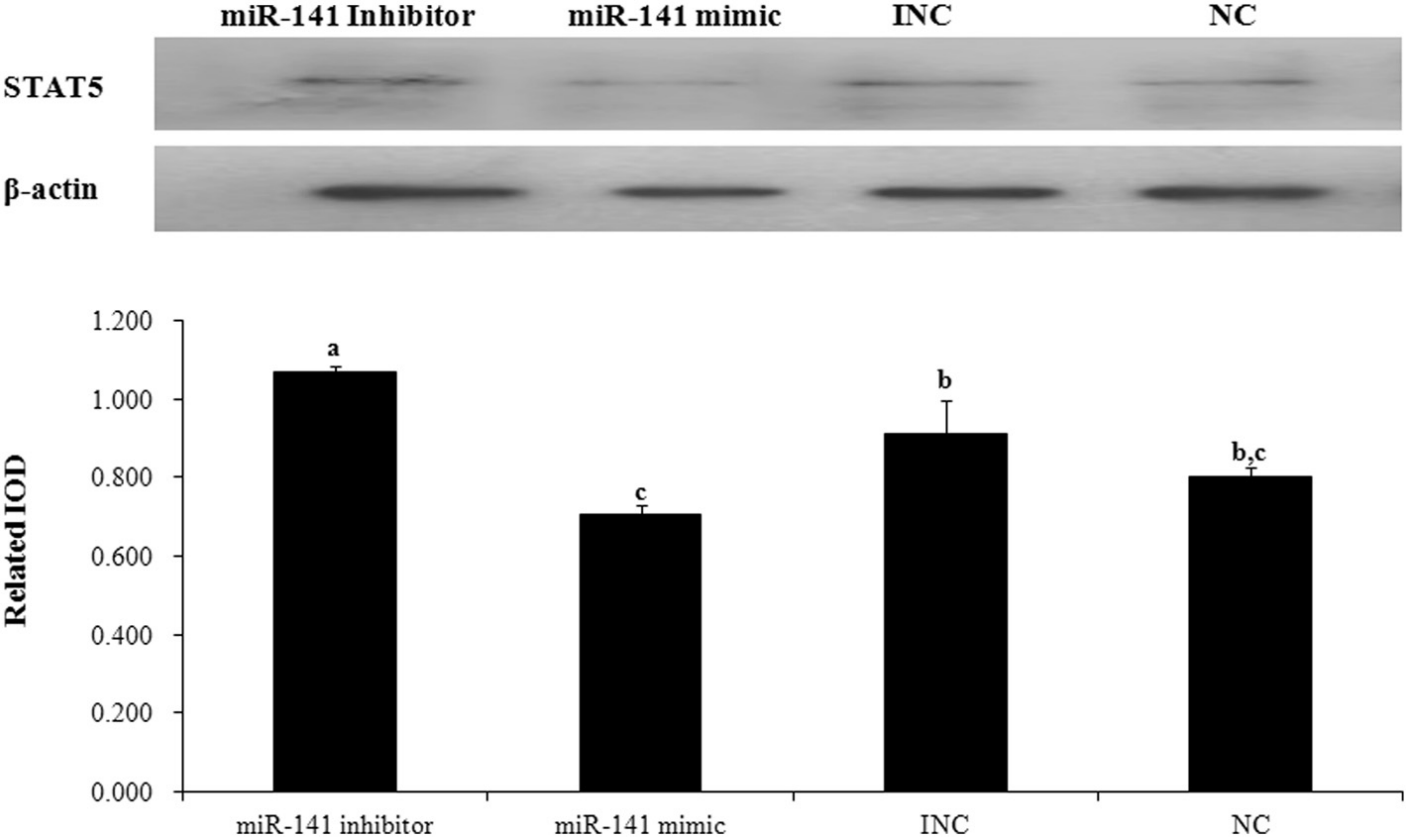
**STAT5 protein is regulated by miR-141.** STAT5 and β-actin protein expression was determined by western blotting. Mac-T cells were transfected with an miR-141 inhibitor, an miR-141 mimic, an inhibitor negative control (INC) and a negative control (NC). Cell lysates were collected after 24 hours and analyzed by western blotting. Beta-actin served as the internal control. The protein fragment intensity was quantified using Imagpro-Plus software, and IOD was used to represent the protein level. Error bars represent SD. n=3. ^**a-c**^ means different superscripts differ (*P*<0.05).

MiR-141 mimics target and downregulate STAT5, while miR-141 inhibitors may increase STAT5 protein levels by antagonizing the effect of miR-141. Therefore, it is conceivable that miR-141 could be involved in regulating STAT5 gene function during lactation (Figure 10).

**Figure 11:**
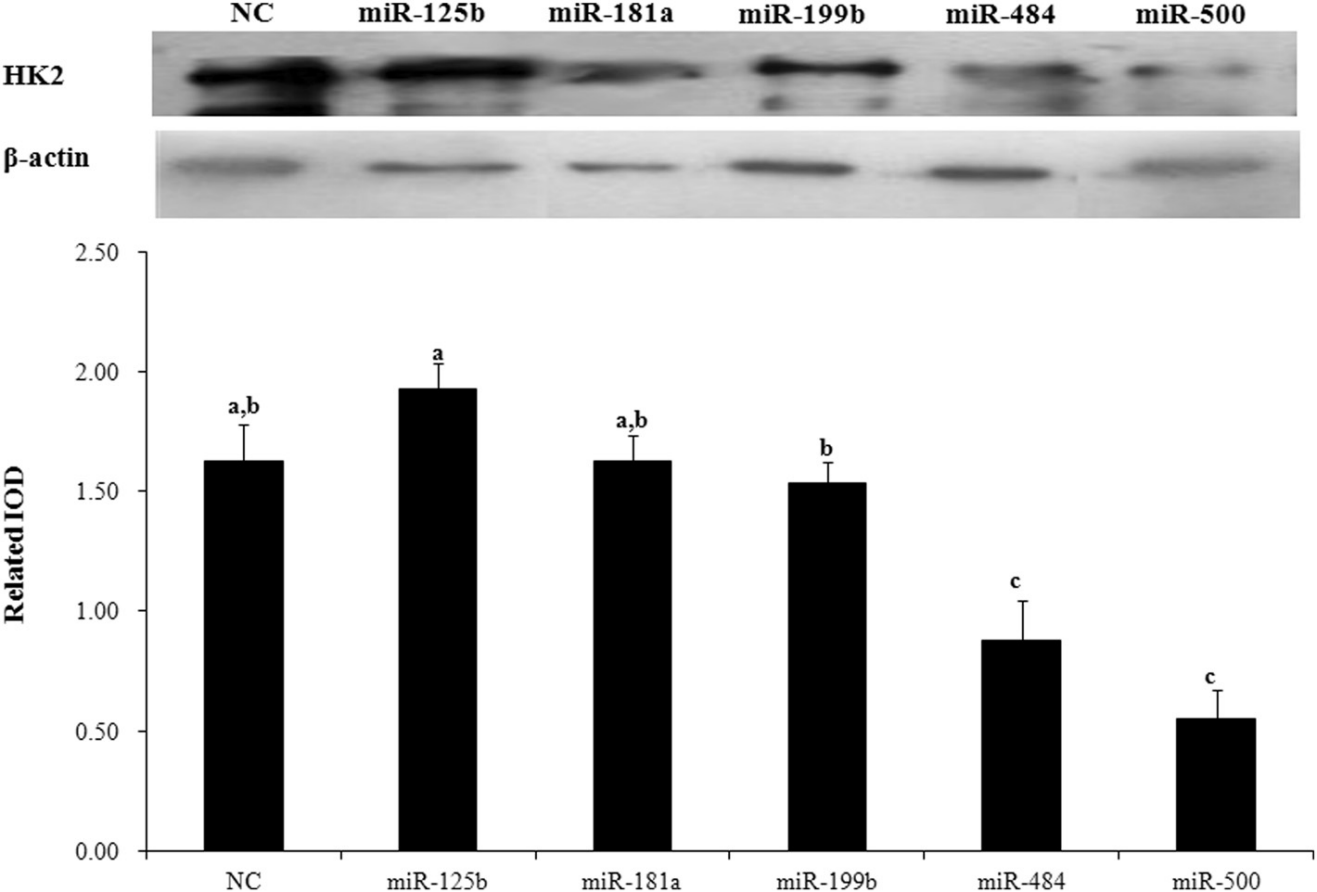
**HK2-targeting miRNAs regulate the expression of HK2 protein. HK2 and β-actin protein expression was determined by western blotting.** Mac-T cells were transfected with HK2-targeting miRNAs and a negative control (NC). Cell lysates were collected after 24 hours and analyzed by western blotting. Beta-actin served as the internal control. The protein fragment intensity was quantified using Imagpro-Plus software, and IOD was used to represent the protein level. Error bars represent SD. n=3. ^**a-c**^ means different superscripts differ (*P*<0.05).

MiR-125b, miR-181a, miR-199b, miR-484 and miR-500 can regulate expression of the *HK2* gene according to our network. To examine the effects of these 5 miRNAs on HK2 expression, Mac-T cells were transfected with miRNA mimics or negative control dsRNA. HK2 protein levels were evaluated by western blotting. Transfection with miR-181a, miR-199b and miR-125b mimics did not significantly change the protein level of HK2 (Figure 11), while miR-484 and miR-500 mimics markedly inhibited the production of HK2 protein. Taken together with the fact that no significant differences were observed at the transcriptional level by real-time PCR (data not shown), these results suggest that miR-484 and miR-500 may function at the translational level.

## Discussion

Although the involvement of miRNAs in human MG has been extensively studied, no systematic work has been conducted on bovine MG. Some bovine miRNAs have been recently identified by computational and direct cloning approaches [12,18-21], but most bovine MG miRNAs have not been identified or functionally studied. In this study, an extensive miRNA profile of lactation and non-lactation bovine MG was created. Two small RNA libraries generated a total of 33.2M sequencing reads, from which 27.9M reads of mappable sequences (15–26 nt) were derived. In total, 283 known miRNAs, 96 conserved miRNAs and 505 novel miRNAs were detected in bovine MGs. A custom-designed microarray assay was performed to confirm these sequenced miRNAs. Seventy-four novel miRNAs and 43 conserved miRNAs were detected using this microarray. We propose that these confirmed miRNAs are novel miRNA candidates in bovine MG.

### Differentially expressed miRNAs in bovine MGs

The comparison of miRNA expression between lactation and non-lactation MG allowed us to identify 32 known miRNAs, 10 conserved bovine miRNAs and 14 novel miRNAs that were significantly differentially expressed between the two groups. Although there is no direct evidence showing the relationships between these miRNAs and MG development, it has been reported that the overexpression of miR-107 can lead to decreased rates of cell division with cell cycle arrest [22] and is related to the metabolism of cellular lipids. Milk lipid synthesis in MG is increased during the lactation period. The processes that take place between lactation and non-lactation resemble an epithelial-to-mesenchyme transition (EMT). The loss of inter-epithelial cell-cell contacts, including loss of the cell adhesion molecule E-cadherin, takes place between the two periods [23]. Graziano Martello and colleagues showed that miR-107 induces EMT in breast cancer cells [24]. Taking these observations together suggests that miR-107 could play important roles in lactation. It has been reported that miR-23b represses duct gene expression by down-regulating transforming growth factor-beta (TGF-β) signaling [25]. Our data showed that the growth of the ductal system became active and miR-23b showed opposite expression patterns in the lactation and non-lactation periods. Therefore, we propose that miR-23b may be involved in MG duct system development.

### MiRNA location and structure

There is evidence that miRNAs are often clustered and sometimes co-expressed from the same primary transcript, leading to the hypothesis that they may share functional relationships [26]. MiRNAs clustered on genome loci are ubiquitous in animals. Characteristics of miRNA clusters have already been reported for many mammals [14,27,28]. Based on the consensus criteria, our results reveal a unique feature. The low percentage of clustering seen in our data may be due to the currently incompletely characterized bovine genome. Each cluster contained 2 to 15 pre-miRNAs. The chromosome with the most clusters is chromosome 21, with 8 clusters. Some miRNA clusters contain different types of pre-miRNA, such as cluster 31, housing miR-338, CN-287 and PC-3065 within chromosome 19 (genomic location of pre-miRNA: 53 057 553–53 057 638). These three pre-miRNAs may act cooperatively.

Our most interesting finding is that there were 104 pairs of known miRNAs and novel candidates with the same pre-miRNA structures but very different counts. One potential reason for this finding is that the highly sensitive deep sequence technology makes it possible to identify novel candidates with lower counts. Another possible explanation is that the pre-miRNA hairpin can be cleaved into two arms. One arm is the mature miRNA, which is loaded with Argonaute (Ago2) proteins into the RNA-induced silencing complex (RISC), where it guides the RISC to silence target mRNAs, whereas the other arm goes on to be degraded via the miRNA processing canonical pathway. Because mature miRNA is derived from different pre-miRNAs depending on the body’s needs, the amount of the degraded arm will vary under different physical conditions.

### Lactation network

MiRNAs exert their effects by interacting with target mRNAs. Therefore, target-predicting software (Targetscan) was used to identify putative targets. On the basis of the predicted targets of the 283 known miRNAs, an interaction network composed of these miRNAs and their candidate targets expressed during lactation was constructed by EGAN. Using this network, it was demonstrated that 37 miRNAs interact with a total of 15 targets, which are involved in amino acid, fatty acid and lactose metabolism. It has been reported that the increased activity of the xanthine dehydrogenase (*XDH*) gene, a putative target of miR-29, miR-15b and miR-107, is an early event in mammogenesis *in vivo* and *in vitro* rather than a terminal component of differentiation [29]. MiR-362, miR-25, miR-363 and miR-32 target *BCAT2* encodes a branched chain aminotransferase found in mitochondria. It has recently been observed that branched chain amino acids can play a signaling role for protein synthesis in addition to serving as substrates [7]. In a study designed to determine the effect of lactation, it was found that *BCAT* activity increased in mammary tissue during rat lactation and was 6-fold higher than in virgin rats [30]. *PRLR* is the putative target of miR-142, miR-23, miR-374b, miR-30a and miR-27b and plays a function in MG development together with prolactin. Prolactin promotes alveolar survival, maintains tight junctions and regulates milk protein and lactose synthesis [31]. *STAT5* serves as a common point in the signal transduction pathways of several lactogenic and galactopoietic hormones in the MG [31-33]. Feuermann and colleagues found that one of the targets of *STAT5* is the miR-17/92 cluster [34]. Gene knockout experiments have demonstrated that the formation of lobular-alveolar structures depends upon the actions of progesterone and prolactin receptors functioning through the STAT5, cyclin D1 and Wnt pathways. Molecular analyses have established that Caveolin-1 (*Cav-1*) abrogates PRL-induced gene expression by sequestering *JAK2*. The loss of both *Cav-1* alleles results in precocious MG development during pregnancy and the concomitant precocious activation of *STAT5*. All of the above indicate that although little is known about the exact functions of these miRNAs, the relationships with their respective target genes indicate potential roles in lactation.

Both *HK2* and *STAT5* are key genes in the constructed network. Five related miRNAs were used to validate the network. STAT5 expression patterns were opposite to those of miR-141 in the MG detected in this study. According to our target predictions, we found that the *STAT5* 3’UTR is paired to miR-141 by a 7-mer seed region. Our transfection assay using Mac-T cells revealed that increased levels of miR-141 could reduce STAT5 protein expression. These findings emphasize a potential role of miR-141 in lactation regulation.

The interaction network predicted that *HK2* is a target of miR-125b, miR-181a, miR-199b, miR-484 and miR-500. Currently, there are no reports on any associations between *HK2* and miRNAs. Although 5 miRNA binding sites were predicted in the *HK2* 3’UTR, only miR-484 and miR-500 at high concentrations resulted in significant reductions in HK2 protein level. It is possible that the network is regulated by other factors or that the three miRNA binding sites are located at the position of the *HK2* 3’UTR [35]. The results of this analysis indicated miR-484 and miR-500 as putative miRNA regulators of *HK2*. Therefore, in this study, we have identified a relatively large number of miRNAs from bovine MG, validated 117 newly isolated miRNAs (43 conserved and 74 novel miRNAs), analyzed their expression and predicted their putative targets. Our results also demonstrated that miR-141, miR-484 and miR-500, characterized by the miRNA-gene regulatory networks, are probably essential for lactation via the targeting of *STAT5* and *HK2*.

## Conclusion

The aim of our work was to examine miRNA expression profiles in bovine MG and to evaluate miRNA functions through the identification of differentially expressed miRNAs in lactation and non-lactation MG. Our identification of novel miRNAs highlights the importance of miRNAs with low abundance and less conservation between species. An interaction network of known miRNAs and their target genes relating to lactation was constructed to postulate the functional roles of miRNAs in the MG. This integrated analysis provides important information that may inspire further experimental investigation into the field of miRNAs and their targets during lactation.

## Methods

### Ethics statement

Experiments were performed according to the Regulations for the Administration of Affairs Concerning Experimental Animals (Ministry of Science and Technology, China, revised in June 2004) and approved by the Institutional Animal Care and Use Committee at Zhejiang University, Zhejiang, China. Animals were allowed access to food and water *ad libitum* under controlled environmental conditions and were humanely sacrificed as necessary to ameliorate suffering.

### Sample preparation

Two multiparous dairy cows were used for miRNA library construction. The first was a 6-year-old cow that had been lactating for 2 months, which was used to make the lactation miRNA library, and the second was a 4-year-old non-lactating, non-pregnant cow, which was used to construct the non-lactation miRNA library.

In the microarray assay, two other multiparous cows were added to each period, and mixed RNA samples were made. The two additional lactation cows had been lactating for 3 and 4 months and were 4 and 5 years old, respectively. The two additional non-lactating, non-pregnant cows were 4 and 5 years old.

Bovine MG tissues were collected and immediately stored in liquid nitrogen until further use. Blocks of MG tissue were fixed in 4% formalin for 48 hours, processed and embedded into paraffin blocks according to routine procedures.

### Histologic examination

The paraffin-fixed blocks were serially sectioned into 8 μm coronal slices and stored at −20°C until further use. For routine histological studies, paraffin sections were stained with hematoxylin and eosin. Hematoxylin-eosin stained sections were analyzed by light microscopy using a Nikon fluorescence microscope (Nikon, Japan).

### Immunofluorescence assay

Alpha-casein was detected in frozen sections by immunofluorescence. Sections were fixed with 4% formaldehyde for 10 minutes. The slides were then rinsed 3 times in PBS for 5 minutes each and blocked for 60 minutes. The blocking solution was replaced by primary antibody solution (1:100, gene Tex, USA), and the samples were incubated overnight at 4°C. The next day, slides were rinsed 3 times in PBS for 5 minutes each. FITC-conjugated secondary antibody (1:200) with DAPI was added, and the slides were incubated for 1 hour at 37°C in the dark, followed by 3 rinses in PBS for 5 minutes each. The specimens were viewed under a fluorescence microscope (Nikon, Japan).

### Total RNA isolation, small RNA library preparation and sequencing

Total RNA was extracted using a Qiagen miRNeasy Mini Kit (Qiagen, USA) according to the manufacturer’s protocol. Subsequently, the RNA samples were sent to LC Science (Houston, USA) to construct the small RNA libraries using an Illumina small RNA kit (Illumina, San Diego, USA) and to be sequenced using Genome Analyzer (Illumina, San Diego, USA).

### Sequencing data analysis

Small RNA reads were processed using Illumina’s Genome Analyzer, and the ACGT101-miR program was then used to process the sequencing data. The mammalian miRbase (miRbase 17.0: http://www.mirbase.org/index.shtml) and the bovine mRNA Rfam, Repbase, genome and EST databases (http://www.ncbi.nlm.nih. gov/projects/genome/guide/cow/ and BTA 4.0: ftp://ftp.ensembl.org/pub/release-57/ fasta/bos_Tau-rus/dna/) were exploited. The sequencing data were first filtered into mRNA using Rfam and Repbase, and then mapped to miRbase. The mapped data were then aligned to genome and EST databases for annotation purposes. The remaining unmapped data were mapped to genome and EST data, secondary structures were predicted using UNAFold software [36], and IDEG 6 was used to identify significant differentially expressed miRNAs [37].

All sequencing data were categorized into three groups: (1) known miRNAs reported in miRbase; (2) conserved miRNAs sharing highly similar sequences corresponding to their precursors in other mammalian genome assemblies, and (3) bovine novel candidates where reads and the predicted secondary structures are not mapped to the miRNAs or pre-miRNAs in miRbase, but are mapped to the *bta* genome with extended sequences from the genome that form hairpins.

### Microarray assay

Total RNA was extracted using a Qiagen miRNeasy Mini Kit (Qiagen, USA). For each stage, equal quantities of total RNA isolated from three individual cows were pooled. A custom-designed microarray assay was performed to analyze miRNA expression patterns in lactating and non-lactating periods by LC Science (Houston, USA). The array included probes for 523 miRNA derived from the sequencing data and reported bovine miRNA (from miRbase) with 5S rRNA as a data normalization control. The probes were synthesized by *in situ* synthesis using PGR (photogenerated reagent) chemistry. Hybridization was performed overnight on a μParaflo microfluidic chip using a micro-circulation pump (Atactic Technologies) [38]. Hybridization images were collected using a laser scanner (GenePix 4000B, Molecular Device) and digitized using Array-Pro image analysis software (Media Cybernetics). Data were analyzed by first subtracting the background and then normalizing the signals using a LOWESS (Locally weighted Regression) filter [39].

### Target prediction and network construction

The starting point of the miRNA target prediction strategy was the utilization of known miRNAs listed in Additional file 1. TargetScan (Version 5.0) was used to predict putative targets with an established miRNA seed database and a bovine 3’EST database. EGAN software was used to depict the relationships between miRNAs, target genes and lactation. Due to limited data in cattle, data from human orthologs were also included for these targets in EGAN software [40].

### Quantitative RT-PCR assay

The gene expression assay and differentially expressed miRNAs identified using deep sequencing were validated using real-time PCR. Total RNA were extracted from the MG tissues in both periods separately using Trizol reagent (Invitrogen, USA). The RNA was divided into two portions, one for genetic testing and the other for miRNA detection. Genetic testing started with 500 ng of total RNA, and this RNA was reverse transcribed to cDNA using a SYBR® PrimeScript® RT-PCR Kit (TAKARA, Japan). For miRNA detection, 2 μg of total RNA was reverse transcribed to cDNA with a specific stem-loop primer using M-MLV (Invitrogen, USA), with incubation for 60 minutes at 42°C, followed by heating for 10 minutes at 95°C and storage at 4°C. These cDNA were then used as templates in a SYBR® Premix Ex Taq™ kit (TAKARA, Japan) with specific primers (Additional file 8). Real-time PCR was performed on an ABI7500 system (Applied Biosystem, USA). The reaction mixtures were incubated in a 96-well plate at 95°C for 30 seconds, followed by 40 cycles of 95°C for 5 seconds and 60°C for 34 seconds. All reactions were run in triplicate. *GAPDH* was used as a gene assay control and bovine S18 rRNA as a miRNA control. Fold changes were determined by the threshold cycle (CT). Fold changes of miRNA expression were calculated using the 2^−ΔCt^ method, where ΔCt = (Ct _target_ − Ct _control_) _Sample_.

### Cell culture and transfection

The cells used in this study were from the Mac-T cell line, which was donated by Dr. Zhao (University of Vermont, Burlington, USA). Cells were maintained in Dulbecco's Modified Eagle's Medium (DMEM, Gibco, USA) supplemented with 10% (V/V) fetal bovine serum (FBS, Gibco, USA), 100 U/mL penicillin and 100 mg/mL streptomycin. The cells were maintained at 37°C with 5% CO_2_ and subcultured every other day.

### Transfection of Mac-T cells with mimics and inhibitors

Cells were seeded in a 24-well plate at concentration of 1×10^5^ cells/ml/well on the day before the transfection. Mimics of miR-125b, miR-141, miR-181, miR-199a, miR-484 and miR-500 and the antisense inhibitor miR-141 were transfected by Lipofectamine 2000 (Invitrogen, USA) according to the manufacturer’s protocol. The transfection efficiency was examined using FAM-conjugated siRNA. The mimics were RNA duplexes, the inhibitors were single-stranded, and the negative controls (NC) and inhibitor negative controls (INC) for all miRNA mimics and inhibitors were designed by Invitrogen and had no homology to any bovine genome sequences (Additional file 9). The culture medium was changed 6 hours after the transfection of 20 pmol/L of mimics or inhibitors. All transfection data are representative of three independently repeated transfections and each 3-well group of cells were treated as one experimental unit.

### Western blotting analysis

Proteins were extracted using a protein extraction kit according to the manufacturer’s instructions (Kegen, China). Equal amounts of protein lysate were separated by SDS- polyacrylamide gel electrophoresis (PAGE) and then electrophoretically transferred to polyclonal difluoride membranes. Each protein was incubated with a specific antibody and detected with an electrogenerated chemiluminescence (ECL) kit. Beta-actin was used as a loading control. Antibodies for STAT5 and β-actin were manufactured by Boster (China), and the HK2 antibody was purchased from Santa Cruz (USA). The intensity of the protein fragments was quantified using Imagpro-Plus software. All data are from three independently repeated experiments.

### Statistical analysis

All data were analyzed using SPSS software (V16.0, SPSS Inc., USA). Values in the texts and figures represent the results of at least three separate experiments. Group comparisons were performed using ANOVA with the Student’s t-test. Differences were considered statistically significant at *P*<0.05.

## Abbreviations

Ago2: Argonaute; CAV-1: Caveolin-1; CV: Coefficients of variation; ECL: Electrogenerated chemiluminescence; EMT: Epithelial-to-mesenchyme transition; FBS: Fetal bovine serum; GO: Gene ontology; HE: Hematoxylin-eosin staining; HK2: Hexokinases; IF: Immunofluorescence; INC: Inhibitor negative control; L: Lactation; MG: Mammary gland; miRNA: MicroRNA; NC: Negative control; NL: Non-lactation; nt: Nucleotides; RISC: RNA-induced silencing complex; SDS-PAGE: SDS-polyacrylamide gel electrophoresis; siRNA: Small interference RNA; STAT5: Signal transducers and activators of transcription; TGF-β: Transforming growth factor-beta; XDH: Xanthine dehydrogenase.

## Competing interests

The authors declare no competing interests.

## Authors’ contributions

ZL performed the main experiment and wrote the paper. HL participated in the study design and paper revision. XJ was involved in executing the study. LL assisted with the experimental design and was involved in revising the paper. JL designed the study, guided the execution of the study, and revised the paper. All authors have read the manuscript and approved its publication.

## Supplementary Material

Additional file 1 **Table S1.** Profile of known bovine miRNAs. Profile of the known bovine miRNA. Click here for file

Additional file 2 **Table S2.** Profile of conserved miRNAs originating from pre-miRNAs. Profile of conserved miRNAs originating from pre-miRNAs. Click here for file

Additional file 3 **Table S3.** Profile of bovine novel miRNAs. Profile of bovine novel miRNAs. Click here for file

Additional file 4 **Table S4.** Profile of microarray assay. Profile of microarray assay. Click here for file

Additional file 5 **Table S5.** Differentially expressed miRNAs. Differentially expressed miRNAs. Click here for file

Additional file 6 **Table S6.** Bovine pre-miRNAs with two or more genome locations. Cattle pre-miRNAs with two or more genome locations. Click here for file

Additional file 7 **Table S7.** Same pre-miRNA structure and location. Same pre-miRNA structure and location. Click here for file

Additional file 8 **Table S8.** Primer sequences used in the q-PCR experiments. Primer sequences of the q-PCR experiments. Click here for file

Additional file 9 **Table S9.** Small interfering RNA. Small interference RNA. Click here for file
